# Effect of 2 Forms of Tenofovir on Duodenal Enterocytes—A Hypothesis for Different Effect of Tenofovir Disoproxil Fumarate and Tenofovir Alafenamide on Body Weight and Plasma Lipids

**DOI:** 10.1093/cid/ciae374

**Published:** 2024-07-23

**Authors:** Kai Juhani Kauppinen, Inka Aho, Nelli Sjöblom, Olli Tynninen, Anu Suomalainen, Ursula Schwab, Fang Zhao, Perttu Arkkila, Jussi Sutinen

**Affiliations:** Department of Infectious Diseases, Inflammation Center, Helsinki University Hospital, Helsinki, Finland; Faculty of Medicine, University of Helsinki, Helksinki, Finland; Department of Infectious Diseases, Inflammation Center, Helsinki University Hospital, Helsinki, Finland; Faculty of Medicine, University of Helsinki, Helksinki, Finland; Faculty of Medicine, University of Helsinki, Helksinki, Finland; Department of Pathology, Helsinki University Hospital, Helsinki, Finland; Faculty of Medicine, University of Helsinki, Helksinki, Finland; Department of Pathology, Helsinki University Hospital, Helsinki, Finland; Stem Cells and Metabolism Research Program, Faculty of Medicine, University of Helsinki, Helsinki, Finland; HiLife, University of Helsinki, Helsinki, Finland; HUS Diagnostic Center, Helsinki University Hospital, Helsinki, Finland; School of Medicine, Institute of Public Health and Clinical Nutrition, University of Eastern Finland, Kuopio, Finland; Department of Medicine, Endocrinology and Clinical Nutrition, Kuopio University Hospital, Wellbeing Services County of North Savo, Kuopio, Finland; Faculty of Medicine, University of Helsinki, Helksinki, Finland; Faculty of Medicine, University of Helsinki, Helksinki, Finland; Abdominal Center, Department of Gastroenterology, Helsinki University Hospital, Helsinki, Finland; Department of Infectious Diseases, Inflammation Center, Helsinki University Hospital, Helsinki, Finland; Faculty of Medicine, University of Helsinki, Helksinki, Finland

**Keywords:** tenofovir disoproxil fumarate, tenofovir alafenamide, enterocyte, duodenal villus, intestinal fatty acid–binding protein

## Abstract

**Background:**

Tenofovir disoproxil fumarate (TDF), compared to tenofovir alafenamide (TAF), leads to lower body weight and plasma lipids by an unknown mechanism. We hypothesize that TDF, when absorbed, may damage enterocytes of the proximal duodenum, leading to reduced absorption of nutrients.

**Methods:**

People with human immunodeficiency virus, without significant gastrointestinal symptoms, receiving a regimen containing TDF (n = 12) or TAF (n = 12), underwent esophagogastroduodenoscopies. Plasma/serum concentrations of nutrients absorbed from proximal duodenum and serum intestinal fatty acid–binding protein (I-FABP), a marker of enterocyte damage, were measured. Cytochrome c oxidase/succinate dehydrogenase (COX/SDH) staining and electron microscopy (EM) were conducted to evaluate mitochondria.

**Results:**

Five patients in the TDF group (1 celiac disease [excluded from further analyses], 1 *Helicobacter* gastritis, and 3 esophagitis) and 2 in the TAF group (2 esophagitis) had a pathological finding in esophagogastroduodenoscopy. Villi were flatter (337 [59] vs 397 [42] μm; *P* = .016), crypts nonsignificantly deeper (200 [46] vs 176 [27] μm; *P* = .2), and villus-to-crypt ratio lower (1.5 [0.42] vs 2.5 [0.51]; *P* = .009) in the TDF versus TAF group (mean [standard deviation]). I-FABP concentration was higher in the TDF versus TAF group (3.0 [1.07] vs 1.8 [0.53] ng/mL; *P* = .003). The TDF group had numerically but not statistically significantly lower concentrations of folate and vitamins A, B1, D, and E. COX/SDH staining and EM showed similar mitochondrial damage in both groups.

**Conclusions:**

Duodenal villous alterations may explain TDF-associated decrease in body weight and plasma lipids. Larger studies are needed to evaluate concentrations of nutrients absorbed from duodenum among TDF users..

**Clinical Trials Registration:**

NCT05326971; EudraCT 2022-000849.

## Teaser

People with stable HIV receiving tenofovir disoproxil fumarate (TDF) versus tenofovir alafenamide (TAF) exhibited shorted duodenal villi, decreased villous-to-crypt ratio, and higher plasma I-FABP. These findings may explain the different effect of TDF versus TAF on body weight and plasma lipids.

The increasing prevalence of obesity among people with human immunodeficiency virus (HIV) [1] has raised interest in the effect of individual antiretroviral agents on body weight. Tenofovir alafenamide (TAF) has been associated with weight gain compared to tenofovir disoproxil fumarate (TDF) [2, 3]. However, recent data imply that TAF has neutral effect on body weight whereas TDF suppresses weight gain [4–9]. The mechanism for this remains unknown, although gastrointestinal side effects (nausea, decreased appetite) and mitochondrial toxicity have been suggested [7, 10].

TDF also decreases plasma lipid concentrations when added as a single agent [11], or when compared to TAF among people with HIV (PWH) [12, 13] or among HIV-negative subjects when used for preexposure prophylaxis (PrEP) [5]. Also, low serum folate, phosphate, and calcium concentrations with functional vitamin D deficiency have been reported among users of TDF-containing regimens [14–16]. These metabolic effects of TDF could possibly be explained by decreased absorption of nutrients. After ingestion, TDF is prone to chemical and enzymatic hydrolysis by intestinal esterases, so it is mostly absorbed from the proximal duodenum [17]. TDF is metabolized within enterocytes into free phosphonate tenofovir (TFV) [18]. The effect of TFV in enterocytes is unknown, but in kidney tubular cells TFV can cause significant cellular damage [19, 20]. Dysfunctional enterocytes could lead to reduced absorption of nutrients. Notably, the proximal duodenum is the site of absorption of a considerable proportion of lipids, lipid-soluble vitamins, folate, calcium, phosphate, iron, and other micronutrients [21].

TAF is more stable in biological matrices than TDF and is primarily hydrolyzed into active TFV by lysosomal carboxypeptidase cathepsin A, mostly in lymphoid cells [22, 23]. This and the significantly smaller dose of TAF versus TDF likely lead to a lower accumulation of TFV within enterocytes.

To investigate this hypothesis, we recruited PWH using either TDF or TAF and conducted esophagogastroduodenoscopies to obtain biopsy samples from duodenum for histology and mitochondrial studies. We also measured plasma concentrations of substances absorbed from the proximal duodenum as well as intestinal fatty acid–binding protein (I-FABP), a circulating marker of enterocyte damage. I-FABP increases in various intestinal pathologies; for example, in celiac disease its concentration correlates with severity of villous atrophy [24, 25].

## MATERIALS AND METHODS

### Participants

People with HIV using TDF- or TAF-containing regimens were recruited. The inclusion criteria were age ≥18 years, stable antiretroviral therapy (ART) with TDF or TAF backbone for ≥6 months, and plasma HIV viral load <200 copies/mL for ≥6 months. Exclusion criteria were known or suspected enteropathy (celiac disease, inflammatory bowel disease) and pregnancy, as well as any contraindication to esophagogastroduodenoscopy. The groups were matched for age, sex, and the core ART class (integrase inhibitors, nonnucleosides, protease inhibitors).

### Assessments

Data on body weight and plasma lipid concentrations before starting TDF or TAF were collected from medical records.

At the study visit, blood samples were drawn after an overnight fast. Circulating concentrations of lipids, calcium, iron, folate, β-carotene, and vitamins A, B1, E, and D were analyzed using standard procedures. The participants were asked to pause supplements containing calcium, folic acid, iron, and vitamin A, B, or E for 1 month prior to the visit; vitamin D use was permitted. Serum I-FABP was measured by enzyme-linked immunosorbent assay (Human FABP2/I-FABP, Quantikine ELISA, R&D Systems, Minneapolis, Minnesota).

Body composition was measured by bioelectrical impedance analysis (Tanita MC-980MA, Tanita Corporation, Tokyo, Japan).

Dietary intake was measured by 3-day food records, which were analyzed by AivoDiet dietary intake calculation software (version 2.2.0.0; Mashie FoodTech Solutions Finland Oy, Turku, Finland) by a clinical nutritionist blinded to study groups.

Esophagogastroduodenoscopies were performed by an experienced gastroenterologist blinded to study group allocation. Macroscopic appearance of the esophagus, ventricle, and duodenum was evaluated, and biopsies were collected from the distal and proximal duodenum and the antrum and corpus of the stomach. For enzyme histochemistry, samples were snap-frozen in liquid nitrogen.

For histopathology, 3-μm sections (n = 2) were cut from formalin-fixed, paraffin-embedded specimens and stained with hematoxylin and eosin. An experienced pathologist, blinded to participants’ group allocation, examined the samples. Biopsies were scanned with a Leica GT450 scanner (Leica, Nussloch, Germany) for digital archiving of the results. All villi and crypts that could reliably be evaluated were measured using Neagen's neaLink digital pathology solution (Helsinki, Finland). Villous atrophy was evaluated according to the Modified Marsh-Oberhuber classification; class 3a or greater was regarded as villous atrophy [26].

Cytochrome c oxidase (COX)/succinate dehydrogenase (SDH) histochemical method was performed on 12-μm cryostat sections as previously described [27]. COX activity was assessed semiquantitatively into 3 categories (no COX-deficient epithelial cells, <50% COX-deficient epithelial cells, ≥50% COX-deficient epithelial cells) by an expert in mitochondrial pathology, blinded to study group allocation.

For electron microscopy (EM) studies of the mitochondria, we selected 2 participants from the TDF group with the most severe signs of villous damage (lowest villus-to-crypt [V:C] ratio), and 2 from the TAF group with the most normal villous structure (highest V:C ratio). One HIV-negative duodenal sample was included as a control. Samples were double fixed with 2% glutaraldehyde/1% osmium tetroxide, resin embedded and ultrathin sectioned, and heavy metal electron stained by uranyl and lead. The sections were observed in a blinded fashion under transmission electron microscope JEOL-1400 (JEOL, Tokyo, Japan). Digital electron micrographs were taken by Morada EM camera (EMSIS GmbH, Münster, Germany).

### Statistical Analysis

Descriptive statistics are presented as median with interquartile range (IQR), mean with standard deviation (SD), or frequencies with percentages. Mann-Whitney *U* test or independent-samples *t* test was used for continuous variables, and χ^2^ test for categorical variables. Pearson *r* and Spearman ρ were used for correlation analyses. Two-tailed values of *P* < .05 were considered statistically significant. Statistical analyses were carried out using SPSS version 27 software (IBM SPSS, Inc, Chicago, Illinois).

### Study Oversight

The study was approved by the National Committee on Medical Research Ethics, Helsinki University Hospital, and the Finnish Medicines Agency. It was registered at the European Clinical Trials Database (EudraCT 2022-000849) and ClinicalTrials.gov (NCT05326971). All study subjects provided written informed consent.

## RESULTS

Twelve participants were included in the TDF group and 12 in the TAF group. One participant in the TDF group had modified Marsh-Oberhuber scale grade 3b villous atrophy, positive transglutaminase antibodies, and HLA-DQ2—a haplotype typical with celiac disease. He was diagnosed with celiac disease—not related to TDF—and was excluded from the analyses.

Before starting TDF versus TAF (pre-TDF/TAF values), the mean (SD) body weight was 92.3 (12.9) versus 79.0 (13.1) kg (*P* = .03), total cholesterol (TC) 199 (68.0) versus 185 (39.0) mg/dL (*P* = .5), low-density lipoprotein cholesterol (LDL-C) 130 (54.0) versus 113 (36.0) mg/dL (*P* = .4), and high-density lipoprotein cholesterol (HDL-C) 42.3 (17.4) versus 57.7 (11.7) mg/dL (*P* = .02), and median (IQR) triglycerides (TG) of 105 (82.4–553) versus 97.4 (64.4–123) mg/dL (*P* = .4), respectively.

The baseline characteristics of the study groups are presented in Table 1.

**Table 1.** Baseline Characteristics of the Study Groups

Characteristic	TDF Group (n = 11)	TAF Group (n = 12)	*P* Value
Male sex, No. (%)	11 (100%)	12 (100%)	1.0
Age, y	55 (12)	57 (16)	.8
White race, No. (%)	11 (100%)	12 (100%)	1.0
Body weight, kg	91.1 (13.0)	80.3 (9.2)	**.031**
BMI, kg/m^2^	28.9 (3.9)	24.7 (2.4)	**.005**
Waist circumference, cm	100.2 (11.2)	93.7 (7.9)	.11
Hip circumference, cm	96.7 (7.3)	93.7 (4.5)	.3
Fat percentage, %	23.3 (6.4)	21.5 (3.9)	.4
Current smoker, No. (%)	2 (18%)	4 (33%)	.6
Use of lipid-lowering agents, No. (%)	5 (46%)	5 (42%)	1.0

Statistically significant *P* values are shown in bold. Data are given as mean (standard deviation) unless stated otherwise.

Abbreviations: BMI, body mass index; TAF, tenofovir alafenamide; TDF, tenofovir disoproxil fumarate.

The TDF group had a nonsignificant decrease in body weight (mean [SD]) during TDF treatment (from 92.3 [12.9] to 91.1 [13] kg, *P* = .5, during 3.7 [1.7] years) and TAF group a similar increase during TAF treatment (from 79.0 [13.1] to 80.3 [9.2] kg, *P* = .7, during 5.0 [2.2] years). No participants had any chronic disease or medication (eg, GLP-1 analogues) affecting body weight during TDF/TAF treatment, but 1 participant in the TAF group had undergone major lifestyle changes and lost 19 kg since starting TAF. Despite the small decrease in body weight during TDF treatment, this group remained heavier than the TAF group; there were no other statistically significant differences between the groups at the study visit (Table 1).

HIV-related characteristics are shown in Table 2. The groups were similar concerning the duration of ART or TDF/TAF treatment and the use of core agents.

**Table 2.** Human Immunodeficiency Virus–Related Characteristics of the Study Groups

Characteristic	TDF Group (n = 11)	TAF Group (n = 12)	*P* Value
HIV transmission mode, No. (%)			.2
Male-to-male sexual contact	7 (64%)	11 (92%)	
Heterosexual contact	4 (36%)	1 (8%)	
Time since HIV diagnosis, y	12 (4.9)	14 (8.1)	.5
Total duration of ART, y	10 (5.0)	14 (6.6)	.15
Time since TDF/TAF initiation, y	3.7 (1.7)	5.0 (2.2)	.13
Core ART class, No. (%)			.8
Subjects taking INSTI	7 (64%)	7 (58%)	
Subjects taking NNRTI	4 (36%)	5 (42%)	
Boosting agent, No. (%)			.3
Cobicistat	0 (0%)	1 (8%)	
Ritonavir	0 (0%)	0 (0%)	
CD4 count, 10^6^ cells/L, median (IQR)	920 (598–1297)	599 (435–801)	.13
Subjects with HIV-1 <50 copies/mL, No. (%)	11 (100%)	11 (92%)	.3

Data are given as mean (standard deviation) unless stated otherwise.

Abbreviations: ART, antiretroviral therapy; HIV, human immunodeficiency virus; INSTI, integrase strand transfer inhibitor; IQR, interquartile range; NNRTI, nonnucleoside reverse transcriptase inhibitor; TAF, tenofovir alafenamide; TDF, tenofovir disoproxil fumarate.

Five patients in the TDF group (1 case of celiac disease, 1 case of *Helicobacter* gastritis, and 3 cases of esophagitis) and 2 patients in the TAF group (2 cases of esophagitis) had a pathological macroscopic or histologic finding (*P* = .2).

Duodenal villi were flatter and V:C ratio was lower in the TDF versus TAF group, especially in the proximal duodenum. Crypts tended to be deeper in the TDF group (Table 3). Only the participant with celiac disease fulfilled the criteria for villous atrophy. The number of intraepithelial lymphocytes was normal in all samples (<30 lymphocytes/100 enterocytes).

**Table 3.** Villus Height and Crypt Depth of Proximal and Distal Duodenal Biopsy Samples

Characteristic	TDF Group (n = 11)	TAF Group (n = 12)	*P* Value
Proximal duodenum VH, μm	337 (59)	397 (42)	**.016**
Proximal duodenum CD, μm	200 (46)	176 (27)	.2
Proximal duodenum V:C ratio	1.5 (0.42)	2.5 (0.51)	**.009**
Distal duodenum VH, μm	343 (50)	372 (49)	.2
Distal duodenum CD, μm	188 (18)	171 (24)	.09
Distal duodenum V:C ratio	1.8 (0.38)	2.2 (0.43)	.08

Statistically significant *P* values are shown in bold. Data are given as mean (standard deviation).

Abbreviations: CD, crypt depth; TAF, tenofovir alafenamide; TDF, tenofovir disoproxil fumarate; V:C ratio, villus-to-crypt ratio; VH, villus height.

Mean (SD) serum I-FABP concentration was significantly higher in the TDF versus TAF group (3.0 [1.07] vs 1.8 [0.53] ng/mL; *P* = .003). In correlation analyses, I-FABP was inversely (ρ = −0.351, *P* = .119) and the V:C ratio positively correlated (ρ = 0.323, *P* = .153) with the percentage change in body mass index (BMI) during TDF/TAF treatment. In a sensitivity analysis excluding the outlier participant who had lost 19 kg during TAF treatment, the correlations between percentage change in BMI and I-FABP and V:C ratio were *r* = −0.46, *P* = .041 and *r* = 0.4, *P* = .081, respectively.

Since more participants in the TDF group had macroscopic or histological pathology, we conducted a sensitivity analysis excluding all participants with macroscopic or histological pathology in TDF and TAF groups. The TDF group still demonstrated shorter villi (*P* = .055), deeper crypts (*P* = .040), lower V:C ratio (*P* = .044), and higher I-FABP concentration (*P* = .001) than the TAF group (data not shown).

**Figure 1.**
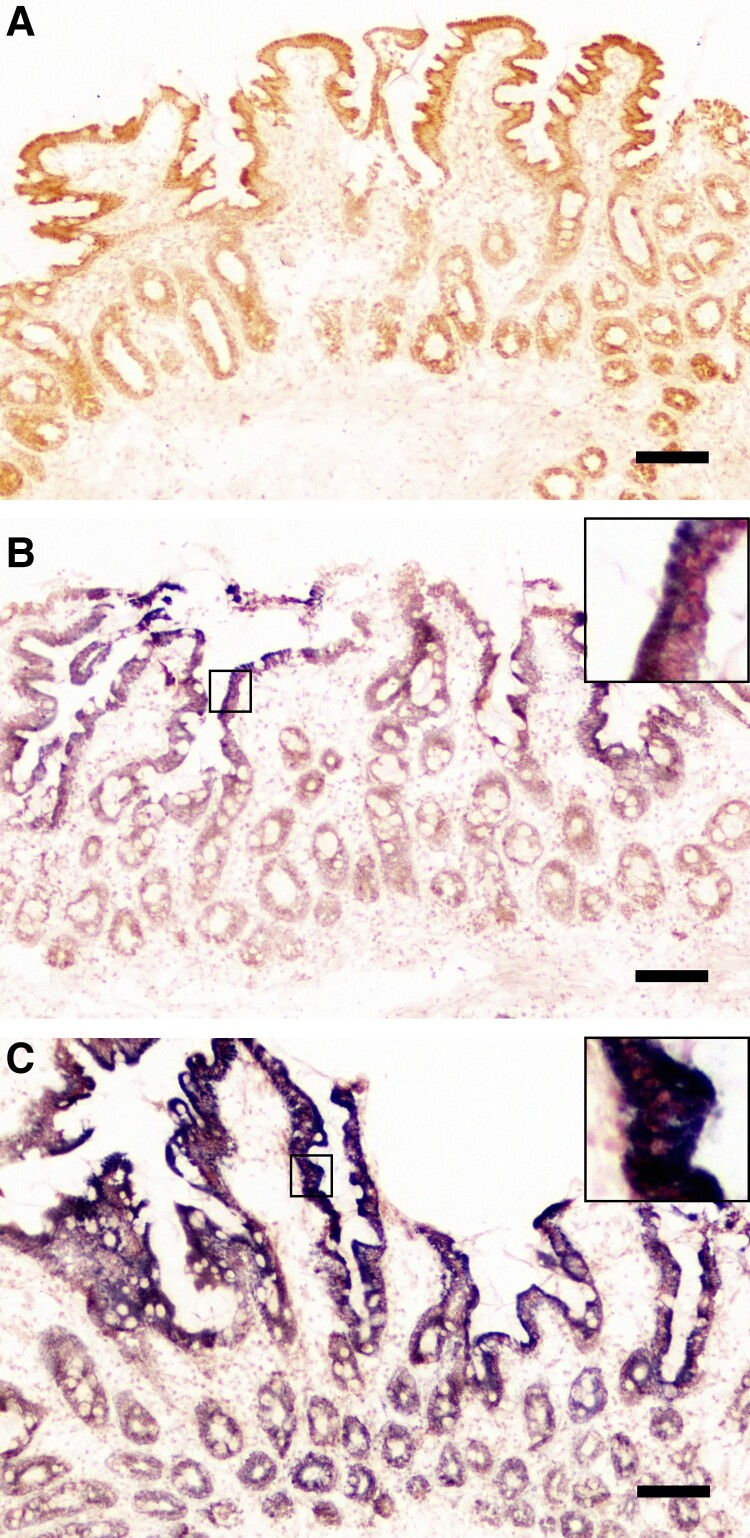
Cytochrome c oxidase (COX) and succinate dehydrogenase double staining of duodenal mucosa. *A*, Normal COX activity in villous epithelial cells and crypts (brown staining). *B*, COX activity is absent in villus epithelial cells (blue staining); <50% of epithelial cells are COX deficient. *C*, COX deficiency in >50% of epithelium of villi and crypts. Scale bar = 100 µm. Insets in *B* and *C* show high-power magnification of COX-deficient epithelium.

In COX/SDH histochemical activity analysis, 10 of 11 participants in the TDF group and 11 of 12 participants in the TAF group (*P* = .9) showed signs of dysfunctional mitochondria of any severity, and 3 of 11 and 6 of 12 (*P* = .265) showed COX deficiency in >50% of epithelial cells in the TDF and TAF group, respectively (Figure 1).

**Figure 2.**
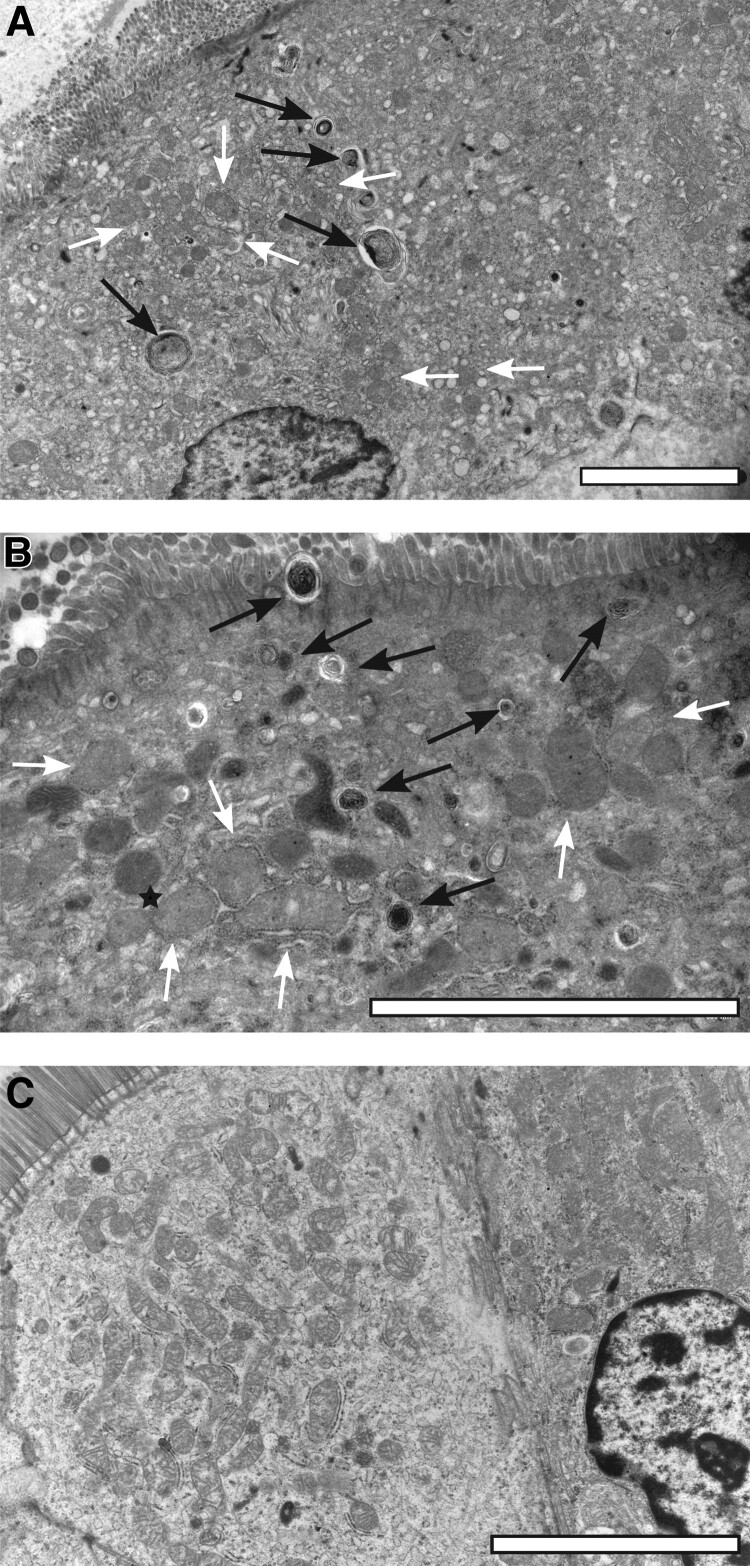
Electron microscopic samples of a participant receiving tenofovir disoproxil fumarate (*A*), a participant receiving tenofovir alafenamide (*B*), and a human immunodeficiency virus–negative control (*C*). Scale bar = 5 µm. Black arrows = myelin figures, white arrows = damaged mitochondria with smudged cristae, black asterisk = mitochondrial fusion.

All EM samples showed similar signs of aberrant mitochondrial structure: changes in mitochondrial size and shape; crista structure changes; and high rate of mitochondrial fusion and high rate of mitophagy manifested by accumulation of lysosomal residue bodies (myelin figures). In addition, dilated rough endoplasmic reticulum (RER), detached ribosomes from RER, disaggregated polyribosomes in the cytoplasm, and abnormal protein misfolding products (aggresomes) were present. The HIV-negative control sample showed no apparent signs of mitochondrial damage, only slight swelling of the mitochondrial matrix (Figure 2).

Ten participants in the TAF group had used TDF previously. The mean time since discontinuation of TDF was 6.7 (SD, 2.0) years. The 2 subjects never exposed to TDF showed signs of mitochondrial toxicity in COX/SDH or EM studies and decreased V:C ratio.

Laboratory test results are shown in Table 4. When compared to pre-TDF/TAF values (mean [SD]), the TDF group had decreased TC (199 [68.0] to 160 [30.0] mg/dL; *P* = .024) and LDL-C (130 [53.6] to 106 [28.9] mg/dL; *P* = .038) concentrations. The TAF group decreased TC statistically significant (185 [39.0] to 165 [35.0] mg/dL; *P* = .04) but LDL-C decrease (113 [35.5] to 100 [34.6] mg/dL; *P* = .3) was not statistically significant. The decrease in HDL-C and TG concentration was not statistically significant in either group.

**Table 4.** Results of the Laboratory Parameters

Parameter^a^	TDF Group (n = 11)	TAF Group (n = 12)	*P* Value
Plasma TC, mg/dL	160 (30.4)	165 (35.4)	.7
Plasma LDL-C, mg/dL	106 (28.9)	100.0 (34.6)	.6
Plasma HDL-C, mg/dL	41.7 (16.4)	56.0 (11.2)	**.022**
Plasma TG, mg/dL, median (IQR)	85.9 (66.4–191.3)	99.6 (84.4–121.8)	.8
Plasma calcium ion, mmol/L, median (IQR)	1.23 (1.19–1.25)	1.21 (1.19–1.25)	.6
Serum 25-hydroxy vitamin D, nmol/L	54 (17)	58 (23)	.8
Plasma iron, μmol/L	17.0 (5.0)	17.1 (7.8)	1.0
Serum folate, nmol/L	20.2 (5.3)	23.7 (7.8)	.2
Serum β-carotene, nmol/L	634 (341)	569 (406)	.7
Serum vitamin A, μmol/L	2.55 (0.59)	2.83 (0.75)	.3
Blood vitamin B1, nmol/L	129.6 (23.4)	145.3 (15.7)	.07
Serum vitamin E, μmol/L	28.7 (6.4)	31.1 (4.6)	.3

Statistically significant *P* values are shown in bold. Data are given as mean (standard deviation) unless stated otherwise.

Abbreviations: HDL-C, high-density lipoprotein cholesterol; IQR, interquartile range; LDL-C, low-density lipoprotein cholesterol; TAF, tenofovir alafenamide; TC, total cholesterol; TDF, tenofovir disoproxil fumarate; TG, triglycerides.

^a^The laboratory samples were collected after an overnight fast.

The concentrations of folate and vitamins A, B1, D, and E were numerically lower in the TDF versus TAF group, yet none of the differences were statistically significant (Table 4).

The results of the food records are presented in Table 5. TDF group consumed more calories (*P* = .039), but not after adjusting for body weight. Cholesterol intake was statistically higher, and consumption of folate and β-carotene as well as vitamins B1, D, and E was numerically higher in the TDF versus TAF group.

**Table 5.** Dietary Intake Based on 3-Day Food Records

Parameter	TDF Group (n = 11)	TAF Group (n = 12)	*P* Value
Energy, kcal, median (IQR)	2276 (1912–2569)	1877 (1682–2467)	**.04**
Energy, kcal/kg body weight, median (IQR)	23.7 (21.5–32.4)	22.2 (20.3–28.4)	.7
Protein, % of energy	20.8 (5.2)	17.0 (3.7)	.06
Protein, g/kg body weight, median (IQR)	1.2 (0.8–1.8)	0.9 (0.8–1.1)	.3
Carbohydrates, % of energy	35.0 (6.4)	47.0 (6.0)	**<.001**
Fat, % of energy	38.4 (7.0)	33.4 (4.5)	.05
MUFA + PUFA, % of total fat intake	56.8 (7.4)	50.7 (7.8)	.07
Cholesterol, mg, median (IQR)	412 (281–865)	230 (174–299)	**.04**
Cholesterol, mg/kg body weight, median (IQR)	4.3 (3.2–10.1)	2.8 (2.3–4.0)	**.04**
Sugars, % of energy	6.9 (2.8)	10.9 (7.3)	.1
Fiber, g	25 (11)	22 (9)	.5
Fiber, g/1000 kcal	11.4 (5.0)	11.4 (4.2)	1.0
Vitamin D, from diet, μg, median (IQR)	8.2 (7.0–12.1)	5.7 (2.5–7.8)	.2
Vitamin D, diet and supplements, μg, median (IQR)	8.2 (7.0–15.0)	6.2 (2.5–28.7)	.2
Vitamin E, mg, median (IQR)	15.0 (8.8–17.5)	10.4 (8.0–13.8)	.2
Vitamin B1, mg	1.7 (0.47)	1.5 (0.52)	.4
β-carotene, mg	12.0 (7.2)	9.8 (7.6)	.5
Folate, μg	335 (110)	226 (127)	.2

Statistically significant *P* values are shown in bold. Data are given as mean (standard deviation) unless stated otherwise.

Abbreviations: IQR, interquartile range; MUFA, monounsaturated fatty acids; PUFA, polyunsaturated fatty acids; TAF, tenofovir alafenamide; TDF, tenofovir disoproxil fumarate.

## DISCUSSION

We have demonstrated that patients receiving TDF as compared to TAF showed signs of duodenal mucosal alterations: Villi were shorter, crypts tended to be deeper, V:C ratio was decreased, and plasma I-FABP concentration—a circulating marker of enterocyte damage—was higher. These data imply that the suppressive effect of TDF on body weight and plasma lipids may be caused by enterocyte dysfunction, leading to decreased absorption of nutrients.

Limited data exist on endoscopic findings among stable PWH without significant gastrointestinal symptoms. In our study, 5 patients (45%) in the TDF group and 2 (17%) in the TAF group had macroscopic or histologic findings, including 1 case of previously undiagnosed celiac disease.

By using a precise digital pathology solution, we observed shorter villi and deeper crypts in the TDF than TAF group. The difference in the V:C ratio was especially pronounced in the proximal duodenum, where absorption of TDF takes place. Villous atrophy, as in celiac disease, is associated with weight loss and decreased plasma lipids [28–30]. Although only the participant with celiac disease had true villous atrophy, it is plausible to presume less effective absorption of nutrients in the affected anatomical site. Interestingly, the angiotensin II receptor blocker olmesartan may cause villous atrophy with weight loss and diarrhea, but its mechanism remains unknown [31, 32].

The TDF group had a higher I-FABP concentration than the TAF group, and I-FABP correlated inversely with the percentage change of BMI during TDF/TAF treatment, suggesting a link between TDF-associated mucosal damage and body weight suppression. Of note, I-FABP is higher among PWH than HIV-negative subjects, and higher among chronic HIV-infected persons than elite controllers; I-FABP has also correlated with viral replication and low CD4 count among PWH [33]. However, none of these factors could explain the difference in I-FABP concentration between our study groups. Direct evidence of TDF increasing I-FABP comes from a study evaluating clearance rates of the anti-HIV monoclonal antibody VRC01 [34]. This study found a significant increase in serum I-FABP after starting TDF-based PrEP and proposed that increased clearance of VRC01 may be due to increased intestinal permeability caused by TDF [34]. TAF-based PrEP was not used in this study.

The data on the mitochondrial effects of TDF and TAF are variable. Neither TDF nor TAF decreased mitochondrial DNA content in human T-cell lines [35]. TAF compared to TDF was linked to reduced cellular respiration and adenosine triphosphate production in peripheral blood mononuclear cells [36]. TDF-associated proximal tubular dysfunction is explained by mitochondrial toxicity, but the effect of TAF on tubular cells is poorly known [20]. We observed similar abnormalities in mitochondrial function (COX/SDH histochemistry) and ultrastructure (EM) in the TDF and TAF groups. Cell/tissue-specific characteristics of mitochondria may explain the differences between enterocytes in our study and blood cell lines or kidney tubular cells reported earlier. Of note, participants for EM studies were selected to present the most and the least severe histological findings, yet the EM findings were similar. Taken together, our data do not support mitochondrial toxicity as the pathophysiological mechanism for duodenal mucosa alterations or suppressed body weight in the TDF group.

In the TAF group, the mean villous height was 397 μm—barely within normal range (400–1500 μm) [37]. Also, 42% of participants in the TAF group had plasma I-FABP concentration above the normal range (≤2.0 ng/mL) [38]. These findings suggest that also TAF, any of the other antiretroviral agents, or HIV infection itself may lead to mucosal alterations, yet significantly less pronounced than caused by TDF. Notably, most participants in the TAF group had used TDF earlier, but the time since its discontinuation was >6 years. Considering that TDF-associated suppression of body weight and plasma lipids is reversed clinically in less than a year after its discontinuation [39, 40], TDF-associated mitochondrial toxicity should have reversed by 6 years if mitochondrial toxicity is the pathophysiological mechanism. Furthermore, both participants with no TDF exposure showed signs of mitochondrial toxicity and decreased V:C ratio. It is therefore unlikely that previous TDF exposure could explain the findings in the TAF group.

According to 3-day food records, the TDF group had a higher intake of energy and cholesterol than the TAF group despite having a decreasing trend in body weight and plasma cholesterol concentration. However, although the difference in cholesterol intake remained statistically significant between the groups after controlling for body weight, the intake of calories did not. These findings, nevertheless, support impaired absorption as the mechanism for the metabolic changes in patients receiving TDF instead of an alternative hypothesis of reduced energy and nutrient intakes due to, for example, nausea or poor appetite.

We aimed to study the absorptive capacity of the proximal duodenum by measuring concentrations of multiple nutrients absorbed from this area. Most vitamin concentrations were numerically lower in the TDF than TAF group, yet the TDF group consumed more of these vitamins. However, the differences between the groups were not statistically nor clinically significant. Of note, our results agree with the earlier observations of PWH frequently having low vitamin D intake and concentration [41, 42].

The limitations of this study include its cross-sectional and nonrandomized design. Another limitation is the difference in the body weight between the groups. The TDF group was heavier than the TAF group before TDF/TAF initiation. Clinicians may have chosen TDF over TAF for overweight patients knowing the weight-suppressing effect of TDF. The tendency for weight loss during TDF and weight gain during TAF treatment, however, confirmed the representativeness of the study groups. Yet the difference in body weight remained significant at the study visit, so its confounding effect cannot be fully excluded. Groups were matched for core ART classes but not for individual ART agents. Of note, no participant received efavirenz, which is known to suppress body weight [10]. The small sample size due to invasive procedures is another limitation. The study may not have been powered to investigate subtle differences—for example, in vitamin concentrations and EM studies. As in all histological 2-dimensional studies, the evaluation of villi and crypts is subject to sample orientation, which we aimed to control for by choosing the most representative sample for each participant. As another limitation, our study population included only White men, which prevented us from investigating potentially different effects on women or people of other ethnicities. The strengths of this study include representative study groups, the possibility to gain direct biopsy samples from the tissue of interest, and analysts being blinded to study group allocation.

To the best of our knowledge, this is the first study to show that ART can affect duodenal mucosa. TDF compared to TAF caused alterations in duodenal villi and crypts, as demonstrated in the biopsy samples, and increased circulating marker of enterocyte damage (I-FABP). This intestinal damage could explain the suppressive effect of TDF on body weight and plasma lipids. These metabolic effects of TDF could be beneficial among PWH with a high prevalence of obesity and dyslipidemia [8], although cohort data on TDF-associated risk for myocardial infarction remain inconclusive [43, 44]. Overall, our study results do not negate these potential metabolic benefits of TDF but call for larger studies to investigate the clinical significance of these duodenal changes on vitamins and other nutrients absorbed from the affected part of the intestine.

## Notes

***Acknowledgments.*** The authors gratefully acknowledge Mia Urjansson for excellent technical assistance (study visits), Anni Kuosmanen (food record processing), Kimmo Kartasalo (figure editing), and Juha Vuorte for bringing up the idea of studying duodenum in this context.

***Author contributions.*** K. J. K.: Study design, data collection and analysis, manuscript writing. I. A.: Study design, interpretation, manuscript writing. N. S.: Study design, histological analyses, manuscript writing. O. T. and A. S.: Study design, mitochondrial analyses (COX/SDH histochemical staining), manuscript writing. U. S.: Study design, food record analyses and interpretation, manuscript writing. F. Z.: Study design, mitochondrial analyses (EM analyses), manuscript writing. P. A.: Study design, esophagogastroduodenoscopies, manuscript writing. J. S.: Study design, data analysis, interpretation, manuscript writing. All authors approved the final version of the manuscript.

***Financial support*.** This work was supported by the Emil Aaltonen Foundation, the Maud Kuistila Memorial Foundation, the Finnish Medical Foundation, the Infectious Diseases Society of Finland, and the Finnish Subsidy for Health Science Research.
